# Analgesic Efficacy Comparison Between Ultrasound-Guided Erector Spinae Plane Block and Retrolaminar Block in Patients Undergoing Modified Radical Mastectomy: A Randomized Controlled Trial

**DOI:** 10.7759/cureus.76124

**Published:** 2024-12-21

**Authors:** Swati Vijapurkar, Sarita Ramchandani, Radhakrishna Ramchandani, Subrata K Singha, Mayank Kumar

**Affiliations:** 1 Anaesthesiology, All India Institute of Medical Sciences, Raipur, Raipur, IND; 2 General Surgery, All India Institute of Medical Sciences, Raipur, Raipur, IND

**Keywords:** dexamethasone, erector spinae plane block (espb), modified radical mastectomy (mrm), retrolaminar plane block (rlb), ropivacaine

## Abstract

Introduction: Breast cancer is the most common cancer in females. Surgery is the gold standard therapy, with modified radical mastectomy (MRM) being the most commonly performed procedure for breast cancer. Management of postoperative pain after MRM poses a clinical challenge and hence receives utmost priority. Ultrasound-guided regional nerve blocks are commonly administered to combat post-surgical pain after MRM. In this context, retrolaminar block (RLB) and erector spinae plane block (ESPB) are relatively newer techniques. Though these blocks are quicker, safer, and easier to administer, very few studies have been done to compare their postoperative analgesic efficacy. Henceforth, we conducted this trial to compare the postoperative analgesic efficacy of ESPB and RLB in patients scheduled for MRM.

Methods: The procedures followed in this trial were according to the norms of the Declaration of Helsinki (2013). The study was started after obtaining approval from the Institutional Ethics Committee (IEC), written informed consent from the patients, and trial registration in the Clinical Trials Registry of India. Sixty female patients of the American Society of Anesthesiologists with physical statuses 1, 2, and 3, aged 18 years and above, planned for unilateral MRM under general anesthesia, were included, whereas patients not giving consent, allergic to study drugs, having contraindications to regional anesthesia, with a body mass index (BMI) of ≥ 35 kg/m^2^, deformity of the spine, psychiatric illness, lactating, or pregnant women were excluded. Computer-generated randomization was used to allocate the patients to groups E and R to receive ESPB or RLB, respectively, using 30 mL of ropivacaine (0.5%) with 2 mL of dexamethasone at the T4 spinous process level under ultrasound guidance. The primary outcome was to determine the time to rescue analgesia based on a visual analog scale score ≥ 4. Secondary objectives were the intraoperative fentanyl consumption and side effects (if any).

Results: Sixty participants completed the study. Both groups were comparable in terms of demographic parameters, duration of surgery, time to the first dose of rescue analgesia, intraoperative fentanyl consumption, and side effects. The mean (SD) of duration of surgery (minutes) was 202.33 (14.55) and 197.00 (18.60) with P = 0.134, time to rescue analgesia (minutes) was 425.67 (134.33) and 468.50 (142.74) with P = 0.236, and intraoperative fentanyl consumption (mcg) was 4.00 (11.02) and 4.67 (8.60) with P = 0.410 in Group E and R, respectively.

Conclusion: Both groups were similar in terms of the time to rescue analgesia, fentanyl consumption intraoperatively, and side effects. Thus, the ESPB is comparable to and not better than RLB for providing postoperative analgesia in patients with carcinoma breast undergoing MRM.

## Introduction

Over the past decade, there has been a steady rise in breast cancer cases. As per the report of the World Health Organization (WHO), 2.3 million newly developed cases of carcinoma breast were diagnosed in women in the year 2020. By the end of the year 2020, a total of 7.8 million cases of women's breast cancer were found to be prevalent in the last five years, making it the most prevalent cancer worldwide. Currently, the most common cancer in Indian females is carcinoma breast [1]. Treatment modalities for breast cancer include surgery, chemotherapy, and radiotherapy. Surgery is considered to be the gold standard treatment, and amongst various surgical procedures, modified radical mastectomy (MRM) is the one that is the most commonly done [2]. Post-operatively, if the pain after breast cancer surgery is not addressed properly, then it may develop into chronic persistent pain [3], increased morbidity, and prolonged hospital stay. An increased incidence of post-mastectomy pain syndrome is noted after MRM [4]. Therefore, treating pain postoperatively after breast cancer surgery receives utmost priority. The inter-costal nerves provide most of the cutaneous innervations of the breast, and the blockade of its nociceptive inputs is essential for post-operative analgesia after breast surgery [5]. Ultrasound (US)-guided regional nerve blocks are commonly administered nowadays to combat post-surgical pain after MRM. Some of the commonly used blocks include pectoral nerve block (PNB), modified PNB, and thoracic para-vertebral block (PVB) [6-8]. Though the use of PVBs for analgesia after breast cancer surgeries has shown satisfactory results, they are associated with certain complications and carry the potential risk of pneumothorax [9]. Retro laminar block (RLB) was introduced as a modified para-vertebral technique in 2006, whereas the erector spinae plane block (ESPB) was put in place later in 2016. Both these blocks are quicker, safer, and easier to administer and have been shown to provide adequate postoperative analgesia after breast cancer surgeries in individual studies [10,11]. However, to date, very few studies have been done comparing the analgesic efficacy of these two blocks.

Henceforth, we conducted this study to compare the postoperative analgesic efficacy of ESPB and RLB in patients of breast cancer undergoing MRM. The primary outcome of our study was to determine the time to the first dose of rescue analgesia in the postoperative period based on a visual analog scale (VAS) score ≥ 4. Secondary objectives were the total dose of fentanyl consumed intraoperatively and side effects (if any).

## Materials and methods

All the procedures in this study were done according to the norms of the Declaration of Helsinki 2013 [12] and the Institutional Ethics Committee (IEC). After obtaining approval from IEC (2032/IEC-AIIMS RPR/2021 dated 30 November 2021) and registration of the trial in the Clinical Trials Registry - India (CTRI/2022/01/039305), this prospective, randomized study was conducted at the All India Institute of Medical Sciences (AIIMS), Raipur, which is a tertiary care center situated in the state of Chhattisgarh in central India from January 2022 till January 2023. Informed and written consent was obtained from all the participants before enrolling them in the study.

Sixty female participants, belonging to the American Society of Anesthesiologists (ASA) physical statuses 1, 2, and 3 [13], aged more than or equal to 18 years, planned for unilateral MRM under general anesthesia (GA) were included in this study, whereas participants not giving consent, allergic to study drugs, having contraindications to regional anesthesia, body mass index (BMI) ≥ 35 kg/m^2^, deformity of the spine, psychiatric illness, lactating, or pregnant patients were excluded.

Computer-generated randomization using the block randomization technique was used to allocate the patients to two groups, E and R, to receive ESPB and RLB, respectively. Random block sizes were used to decrease the selection bias, and the investigator was unaware of the size of each block. Allocation was kept secret by concealing it in a Sequentially Numbered Opaque Sealed Envelope (SNOSE) that was opened by the anaesthesiologist before performing the block inside the operation room (OR), thus keeping the patients blind to the allocated group.

Guidelines issued by the Consolidated Standards of Reporting Trials [14] were followed for the conduct of this study. Study details were explained to the participants one day before the procedure. When the patients arrived in the OR, standard ASA monitors were attached, and recording of the baseline vital parameters, including systolic blood pressure (SBP), diastolic blood pressure (DBP), heart rate (HR), and oxygen saturation of arterial blood (SpO_2_), was done.

Under all aseptic precautions, with the patient being seated and after negative aspiration of blood, either ESPB or RLB was administered under ultrasound guidance using a high-frequency probe (6-13 MHz), as per the group allocation at T4 spinous process level with 30 mL 0.5% ropivacaine and 2 mL dexamethasone. The time of administration of the block was recorded and considered as '0' hours. For the administration of GA, the standard protocol practiced at the institute was followed. All the patients were induced using a 2 mg/kg dose of intravenous (IV) propofol and 0.1 mg/kg of IV morphine. Intravenous vecuronium 0.1 mg/kg was used for the insertion of endotracheal (ET) tube. Intraoperatively, along with isoflurane, a gas mixture of oxygen and nitrous oxide in a ratio of 50:50 was used to maintain anesthesia. For analgesia and muscle relaxation, boluses of IV fentanyl 20 mcg and IV vecuronium 1 mg, respectively, were used intermittently to maintain BP and HR within 20% of baseline readings. Hemodynamic monitoring was performed throughout the surgery, and vital parameters of the patients were noted at every 10-minute interval till the end of the surgery. Reversal of the residual neuromuscular blockade was achieved using 0.05 mg/kg of IV neostigmine and 0.01 mg/kg of IV glycopyrrolate.

Intraoperative complications (if any) such as hypotension (defined as a 20% reduction in SBP as compared to baseline reading), bradycardia (HR less than 50 beats per minute), hypoxemia (defined as SpO_2_ ≤ 94%), local anesthetic systemic toxicity (LAST), allergy to study drugs, etc. were recorded and taken care of as per the standard practice being followed at the institute. The total dose of fentanyl consumed intraoperatively and the duration of surgery were noted.

After extubation, patients were transferred to the post-anesthesia care unit (PACU). In the PACU, standard ASA monitors were attached, and hemodynamic monitoring was continued. Using a VAS of zero to 10 cm, patients were evaluated for pain at rest. A VAS score of zero was considered as having no pain, whereas a score of 10 was considered as having the worst imaginable pain [15]. Assessment of ‘pain at rest’ using the VAS score was done hourly for the first six hours, second hourly till 12 hours, and then at a 24-hour time point from the time of administration of the block. Postoperatively, on the report of a VAS score ≥ 4, IV paracetamol (PCM) 1 gm was administered as the first rescue analgesic. Time to the administration of the first rescue analgesic was noted, starting from ‘0’ hours. Thereafter, IV PCM was administered regularly at eight-hour intervals for the next 24 hours. If patients still complained of pain with VAS ≥ 4, then IV diclofenac 75 mg was administered as an infusion over 30 minutes as a second rescue analgesic. Any side effects that occurred within 24 hours postoperatively, starting from '0' hours, were noted and taken care of as per the standard institutional practice. Microsoft 365 (Microsoft Corporation, Redmond, WA) was used to record the data. Data analysis was done with the help of Statistical Product and Service Solutions (SPSS, version 3; IBM SPSS Statistics for Windows, Armonk, NY).

Continuous and categorical data were represented as mean (standard deviation)/median (interquartile range) and number (n)/percentage (%), respectively. Data were presented graphically wherever found applicable. For continuously distributed data, the independent sample t-test was used, whereas, for the data that were non-normally distributed, appropriate non-parametric tests such as the Wilcoxon test were applied for the comparison of groups. The chi-squared test was applied for categorical data, but if the expected frequency in the contingency tables was less than five or more than 20% of the cells, then Fisher's exact test was used for group comparison. Statistical significance was kept at P < 0.05.

The sample size was calculated based on the standard deviation (SD) and (𝛿) minimum difference between means taken from a previous study done by Zhao et al. in 2021 [16] using the following equation:

n=2 × (𝑍𝛼+𝑍𝛽)2 × σ2 / 𝛿2

where

n = sample size; 𝑍𝛼 = 1.96 at 95% of confidence interval; 𝑍𝛽 = 0.84 at 80% of power; σ = combined SD (1.3); 𝛿 = difference between the means (0.7)

After the substitution of values, a sample size of 54 was derived, with 27 participants per group. Considering dropouts to be 10%, another three cases were added to individual groups, and a final sample size of 60 was derived with 30 participants in each group.

## Results

**Figure 1:**
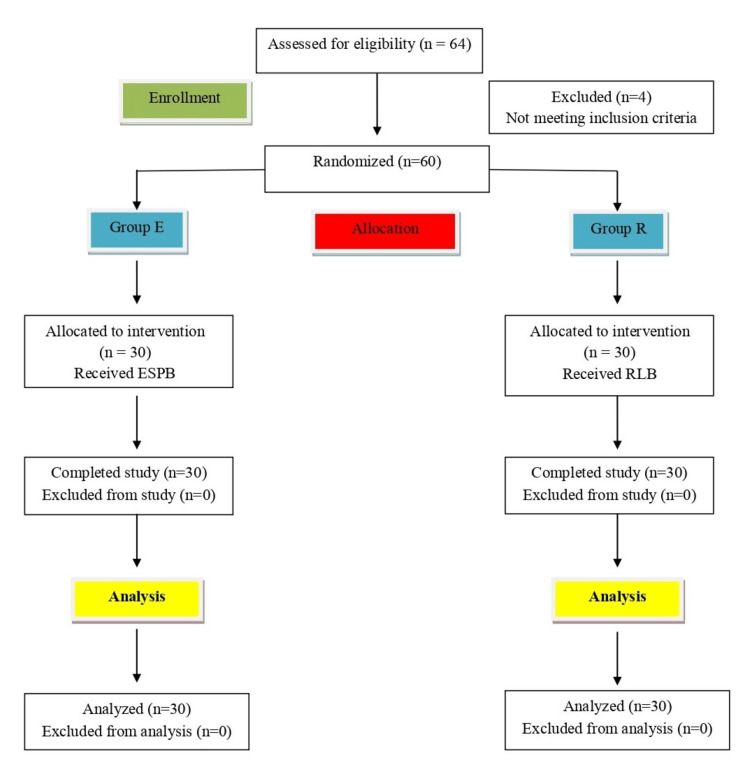
Consolidated Standards of Reporting Trials (CONSORT) flow chart

**Figure 2:**
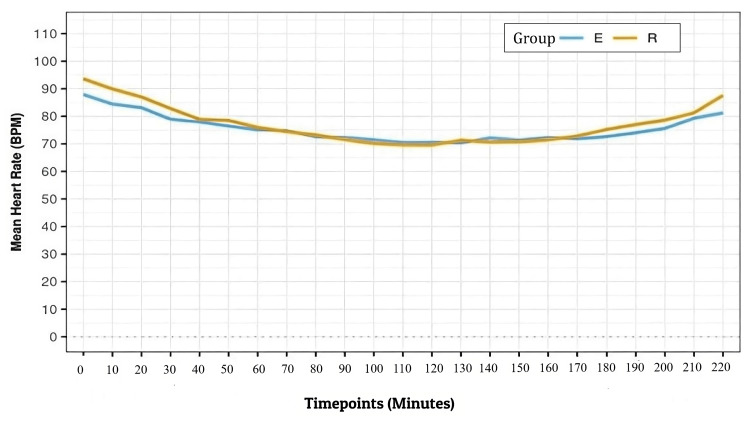
Line diagram depicting the change in mean HR (BPM) over time during the intraoperative period in the two groups HR = Heart rate, BPM = Beats per minute, E = Erector spinae plane block, R = Retrolaminar plane block

**Figure 3:**
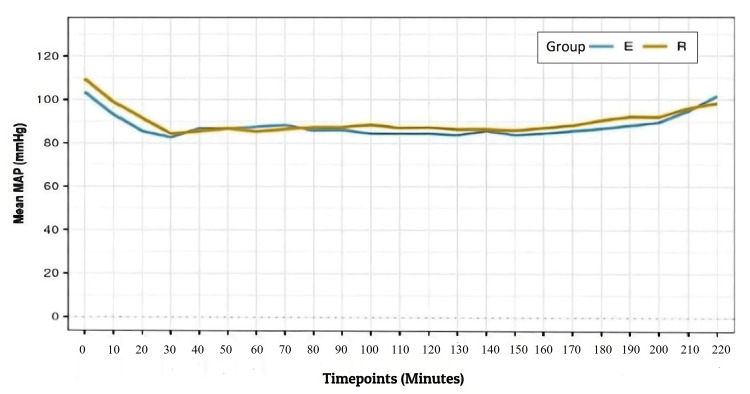
Line diagram depicting the change in mean MAP (mmHg) over time during the intraoperative period in the two groups MAP = Mean arterial pressure, E = Erector spinae plane block, R = Retrolaminar plane block

Sixty eligible patients completed the study and entered the final analysis (Figure 1). The mean (SD) of HR in beats per minute and the mean (SD) of MAP (mmHg) over various time points intraoperatively were comparable between the groups with P > 0.05 (Figures 2, 3).

The demographic parameters of the patients were similar in both groups (Table 1).

**Table 1:** Demographic and clinical parameters Data are represented as mean (standard deviation) and number (percentage). BMI means body mass index, n = number, % = percentage, E = erector spinae plane block group, R = retrolaminar block group. A p-value less than 0.05 is considered statistically significant. 1 = Wilcoxon-Mann-Whitney U test, 2 = t-test, 3 = Fisher's exact test

Parameters	Group		P value
	E (n = 30)	R (n = 30)	
Age (years)	51.17 (8.87)	48.37 (11.10)	0.133^1^
BMI (kg/m²)	24.89 (2.89)	23.71 (2.16)	0.093^1^
Duration of surgery (minutes)	202.33 (14.55)	197.00 (18.60)	0.134^1^
Intraoperative fentanyl consumption (mcg)	4.00 (11.02)	4.67 (8.60)	0.410^1^
Time to rescue analgesia (minutes)	425.67 (134.33)	468.50 (142.74)	0.236^2^
Intraoperative hypotension (yes)	3 (10.0%)	3 (10.0%)	1.000^3^
Intraoperative bradycardia (yes)	1 (3.3%)	1 (3.3%)	1.000^3^

The mean (SD) of HR in beats per minute and the mean (SD) of MAP (mmHg) over various time points intraoperatively were comparable in both groups with P > 0.05 (Figures 2, 3).

A Wilcoxon-Mann-Whitney U test was used to compare the duration of surgery between the groups. Duration of surgery (minutes) expressed as mean (SD) was 202.33 (14.55) and 197.00 (18.60) with P = 0.134 in groups E and R, respectively (Table 1). The duration of surgery (minutes) ranged from 180 to 240 and 170 to 250, with the median interquartile range (IQR) of 200 (190-210) and 200 (180-207.5) in groups E and R, respectively. Thus, the groups were equivalent in terms of duration of surgery. The Wilcoxon-Mann-Whitney U test was used to compare intraoperative fentanyl consumption between the groups. Total intraoperative fentanyl consumption (mcg) expressed as mean (SD) was 4.00 (11.02) and 4.67 (8.60) in groups E and R, respectively (P = 0.410). Thus, the groups were similar in terms of consumption of fentanyl intraoperatively (Table 1). A parametric t-test was used to compare time to rescue analgesia between the groups. The mean (SD) of time to rescue analgesia (minutes) was 425.67 (134.33) and 468.50 (142.74), with P = 0.236 in groups E and R, respectively (Table 1). Time to rescue analgesia (minutes) ranged from 195 to 790 and 170 to 800, with a median (IQR) of 420 (330 to 497.5) and 450 (386.25 to 532.5) in groups E and R, respectively. Thus, the groups were equivalent in terms of time to rescue analgesia in minutes (Table 1). Fisher's exact test was used to explore the association between the groups and the incidence of side effects. No significant difference was noted between the groups in terms of hypotension, bradycardia, vomiting (P = 1.000), and pruritus (P = 0.492). Thus, the incidence of side effects was comparable in both groups (Table 1). No other complications were noted in either of the groups during the study.

## Discussion

This prospective, randomized controlled trial was conducted to compare the analgesic efficacy of ESPB and RLB in breast cancer patients undergoing MRM. Though some studies have demonstrated the superior efficacy of ESPB compared to GA alone, very few clinical studies have been done to date comparing the efficacy of ESPB and RLB. In a systematic review and meta-analysis by Leong et al. [17], the authors evaluated the analgesic efficacy of ESPB in breast surgery. They noted a significant reduction in postoperative opioid consumption and pain scores following ESPB as compared to GA alone. In a similar study done by Zhang et al. [18], the authors found that USG-guided ESPB was more effective in reducing opioid consumption and pain scores within the first 24 hours postoperatively as compared to GA alone. In another such review conducted by Li et al. [19] in patients undergoing breast surgery, the authors noted significant alleviation of pain and reduction in opioid consumption following ESPB.

Previous anatomical studies that aimed to demonstrate the mechanisms of ESPB and RLB have failed to reach any consensus. Adhikary et al. [20] did a study to compare the spread of injectate after RLB and ESPB in fresh cadavers and noted the additional spread of the injectate to intercostal (IC) space in ESPB. The authors believed that it may contribute to its wider analgesic coverage than the RLB. Ivanusic et al. [21] did a cadaveric study simulating ESPB using 20 mL of methylene blue dye and demonstrated that the dye did not spread to the PV space. Damjanovska et al. [22] conducted a cadaveric study to investigate the volume dependence and spread of injectate from the RL to the PV space. The authors noted the spread of injectate to the PV space in all the specimens when 30 mL volume of injectate was used (high-volume group) as compared to no spread to the PV space when only 10 mL volume was used (low-volume group). Thus, the authors concluded that injectate spread from the RL space to the PV space is strongly volume-dependent. Yang et al. [23] did a cadaveric study to investigate the pattern of dye spread following USG-guided RLB and ESPB by injecting 20 mL of dye solution at the fifth thoracic level. The authors noted a more lateral spread of injectate following ESPB than RLB. Onishi et al. [24] did a review to describe the features and anatomical differences between ESPB and RLB. They found that, in comparison to RLB, the pattern of distribution of injectate was deeper and lateral in ESPB. The authors believe that this could be because of the blocking of IC nerves along with their lateral cutaneous branches in ESPB. The authors stated that the optimal dose and concentration of local anesthetics for these two blocks remain unclear, which needs further investigation with high-quality randomized trials to evaluate their clinical efficacy.

Thus, based on the findings of some previous cadaveric studies, we hypothesized that ESPB would be more efficacious than RLB for providing postoperative analgesia after MRM because of a wider and more lateral distribution of drugs in ESPB [24]. However, contrary to our expectations, on comparison, ESPB was found to be similar and not better than RLB in terms of time to administer the first dose of rescue analgesic when these blocks were administered at the T4 spinous process level using 30 mL of drug. Furthermore, the groups were similar in terms of intraoperative consumption of fentanyl and the occurrence of side effects. The findings of our study were comparable to the study done by Sotome et al. [25], in which the authors used 20 mL of 0.375% levobupivacaine for comparison of analgesic efficacy of RLB and ESPB after breast surgery and found RLB to be comparable to ESPB in terms of analgesic efficacy, intraoperative remifentanil consumption, and occurrence of postoperative nausea and vomiting (PONV).

In our study, a 30 mL volume of LA was used to administer the blocks, which failed to demonstrate the superiority of ESPB over RLB for providing postoperative analgesia in patients undergoing MRM. This could be because a 30 mL volume of drug used for ESPB still might not be sufficient to produce a lateral spread for blockage of IC nerves.

In our study, the time to administer the first rescue analgesic (h) ranged widely from 3.25 to 13.17 in the ESPB and 2.83 to 13.33 in the RLB group. These findings were comparable to the observations of Sotome et al. [25], in which the time to the first rescue analgesic (h) ranged from 2.7 to 24 and 3.0 to 24 in the ESPB and RLB groups, respectively. Dautzenberg et al. [26] did a study in cadavers to see the spread of injectate following ESPB and found a variable pattern of the spread. Though following RLB, the path of the injectate is not yet clear, and the variable spread of injectate following ESPB might explain the broad range of time elapsed to administer the first dose of rescue analgesia. Future studies may be undertaken using more than 30 mL volume of the LA to detect any clinically relevant difference in the analgesic efficacy of these two blocks.

Limitation

Our study was limited in the sense that it was based on a homogenous population, comprising only female patients with carcinoma breast, scheduled to undergo unilateral MRM. Therefore, the findings of our study may not apply to male breast cancer patients or those scheduled for bilateral MRM. Additionally, our study was done at a single tertiary care center. Multi-centric studies might have given different results.

## Conclusions

In this study, both groups were found to be equivalent as far as time to the administration of the first dose of rescue analgesia and intraoperative fentanyl consumption were concerned. The groups were also equivalent regarding the incidence of side effects. Therefore, erector spinae plane block is comparable and not superior to retro laminar block for providing postoperative analgesia in patients with carcinoma breast undergoing MRM.

## References

[1] Malvia S, Bagadi SA, Dubey US, Saxena S (2017). Epidemiology of breast cancer in Indian women. Asia Pac J Clin Oncol.

[2] Riis M (2020). Modern surgical treatment of breast cancer. Ann Med Surg (Lond).

[3] Gärtner R, Jensen MB, Nielsen J, Ewertz M, Kroman N, Kehlet H (2009). Prevalence of and factors associated with persistent pain following breast cancer surgery. JAMA.

[4] Turan M, Karaman Y, Karaman S, Uyar M, Gonullu M (2014). Postoperative chronic pain after breast surgery with or without cancer: follow up 6 months: 14AP1-7. Eur J Anaesthesiol.

[5] Woodworth GE, Ivie RM, Nelson SM, Walker CM, Maniker RB (2017). Perioperative breast analgesia: a qualitative review of anatomy and regional techniques. Reg Anesth Pain Med.

[6] Moller JF, Nikolajsen L, Rodt SA, Ronning H, Carlsson PS (2007). Thoracic paravertebral block for breast cancer surgery: a randomized double-blind study. Anesth Analg.

[7] Goswami S, Kundra P, Bhattacharyya J (2017). Pectoral nerve block1 versus modified pectoral nerve block2 for postoperative pain relief in patients undergoing modified radical mastectomy: a randomized clinical trial. Br J Anaesth.

[8] Kulhari S, Bharti N, Bala I, Arora S, Singh G (2016). Efficacy of pectoral nerve block versus thoracic paravertebral block for postoperative analgesia after radical mastectomy: a randomized controlled trial. Br J Anaesth.

[9] Pace MM, Sharma B, Anderson-Dam J, Fleischmann K, Warren L, Stefanovich P (2016). Ultrasound-guided thoracic paravertebral blockade: a retrospective study of the incidence of complications. Anesth Analg.

[10] Yao Y, Li H, He Q, Chen T, Wang Y, Zheng X (2019). Efficacy of ultrasound-guided erector spinae plane block on postoperative quality of recovery and analgesia after modified radical mastectomy: randomized controlled trial. Reg Anesth Pain Med.

[11] Murouchi T, Yamakage M (2016). Retrolaminar block: analgesic efficacy and safety evaluation. J Anesth.

[12] World Medical Association (2013). World Medical Association Declaration of Helsinki: ethical principles for medical research involving human subjects. JAMA.

[13] Mayhew D, Mendonca V, Murthy BV (2019). A review of ASA physical status - historical perspectives and modern developments. Anaesthesia.

[14] Schulz KF, Altman DG, Moher D (2010). CONSORT 2010 statement: updated guidelines for reporting parallel group randomised trials. BMJ.

[15] Delgado DA, Lambert BS, Boutris N, McCulloch PC, Robbins AB, Moreno MR, Harris JD (2018). Validation of digital visual analog scale pain scoring with a traditional paper-based visual analog scale in adults. J Am Acad Orthop Surg Glob Res Rev.

[16] Zhao Y, Tao Y, Zheng S, Cai N, Cheng L, Xie H, Wang G (2022). Effects of erector spinae plane block and retrolaminar block on analgesia for multiple rib fractures: a randomized, double-blinded clinical trial. Braz J Anesthesiol.

[17] Leong RW, Tan ES, Wong SN, Tan KH, Liu CW (2021). Efficacy of erector spinae plane block for analgesia in breast surgery: a systematic review and meta-analysis. Anaesthesia.

[18] Zhang Y, Liu T, Zhou Y, Yu Y, Chen G (2021). Analgesic efficacy and safety of erector spinae plane block in breast cancer surgery: a systematic review and meta-analysis. BMC Anesthesiol.

[19] Li HF, Shen QH, Zhou XY, Shen X (2021). Analgesic effects of erector spinae plane block for patients after breast surgery: a systematic review and meta-analysis. J Int Med Res.

[20] Adhikary SD, Bernard S, Lopez H, Chin KJ (2018). Erector spinae plane block versus retrolaminar block: a magnetic resonance imaging and anatomical study. Reg Anesth Pain Med.

[21] Ivanusic J, Konishi Y, Barrington MJ (2018). A cadaveric study investigating the mechanism of action of erector spinae blockade. Reg Anesth Pain Med.

[22] Damjanovska M, Stopar Pintaric T, Cvetko E, Vlassakov K (2018). The ultrasound-guided retrolaminar block: volume-dependent injectate distribution. J Pain Res.

[23] Yang HM, Choi YJ, Kwon HJ, O J, Cho TH, Kim SH (2018). Comparison of injectate spread and nerve involvement between retrolaminar and erector spinae plane blocks in the thoracic region: a cadaveric study. Anaesthesia.

[24] Onishi E, Toda N, Kameyama Y, Yamauchi M (2019). Comparison of clinical efficacy and anatomical investigation between retrolaminar block and erector spinae plane block. Biomed Res Int.

[25] Sotome S, Sawada A, Wada A, Shima H, Kutomi G, Yamakage M (2021). Erector spinae plane block versus retrolaminar block for postoperative analgesia after breast surgery: a randomized controlled trial. J Anesth.

[26] Dautzenberg KH, Zegers MJ, Bleeker CP, Tan EC, Vissers KC, van Geffen GJ, van der Wal SE (2019). Unpredictable injectate spread of the erector spinae plane block in human cadavers. Anesth Analg.

